# Research on Safety Interlock System Design and Control Experiment of Combined Support and Anchor Equipment

**DOI:** 10.3390/s22166058

**Published:** 2022-08-13

**Authors:** Pengyu Wang, Guoyong Su, Wenlong Yang, Peng Jing

**Affiliations:** State Key Laboratory of Mining Response and Disaster Prevention and Control in Deep Coal Mines, Anhui University of Science and Technology, Huainan 232001, China

**Keywords:** combined support and anchor equipment, safety interlock system, feedback control, safety interlock control

## Abstract

In view of the risk of collision with humans or equipment arising from a lack of protection in the operation process of combined support and anchor equipment on the heading face, this paper designs a safety interlock system for combined support and anchor equipment. Firstly, a mathematical model of hydraulic power system control and a valve control system based on feedforward–feedback optimization were established according to the power demand of the combined support and anchor equipment. Secondly, according to the reliability indexes of the safety interlock system, corresponding sensor, logic control and execution modules were designed. Ultrasonic sensor groups were arranged at the key positions of the combined support and anchor equipment to capture the position information in real time when the equipment was moving. Thus, the pump-valve hydraulic system was controlled through closed-loop feedback. The experimental results show that the safety interlock system of the combined support and anchor equipment can adjust the revolving speed of the permanent magnet synchronous motor (PMSM) in real time according to the distance from the obstacle, so as to control the pump outlet flow, and then perform interlocking safety control of the hydraulic cylinder’s movement speed. The system can effectively prevent damage to the surrounding equipment or personnel arising from equipment malfunction.

## 1. Introduction

With the development of coal mining technology, mining speed on the working face is becoming faster and faster, and most mines in China face a tension between mining and drivage [1,2]. The drivage speed plays a decisive role in coal mining but is largely limited by the operational safety of supporting, bolting and transportation links [3]. The anchor support device is mainly composed of support equipment and anchor drilling equipment, which can achieve the coordinated and efficient operation of tunneling, anchor drilling and support at the same time, mainly including crawler-type, wheel-type and step-type walking modes. However, due to a lack of safety monitoring and early warning monitoring during operation, equipment collision damage and casualties can occur. In order to improve drivage speed and operation safety, domestic and international researchers have designed drivage, bolt and support equipment of various structures to solve “mining–drivage imbalance” and “drivage–support imbalance” [4,5]. Based on the ACP theory—an excavation support anchor intelligent control theory—Yang et al. proposed the anticollision and cooperative operation of tunneling, support, and anchor equipment in the complex environment of the roadway [6]. Zhang et al. used a virtual environment development platform and the method of combining virtual ray and bounding box to achieve collision detection and early warning monitoring between tunneling and anchor support equipment [7]. Javaid et al. studied effective channel modeling, visible light communication characteristics and underground communication structure in the harmful and unpredictable environment of underground coal mines [8,9,10]. In view of the complex working environment and multiple safety hazards on the heading face, this paper designs the sensor module, logic control module and execution module of a safety interlock system and performs a safety interlock control experiment for the purpose of improving the safety properties of support and anchor equipment.

## 2. Design of a Sensor Module for Safety Interlock System

At present, complex equipment for industrial safety production is usually self-protected by building safety interlock systems. A safety interlock system is mainly composed of a sensor module, a logical solution module, and an executor module [11], with functions such as environment perception, signal processing, logical judgment, and decision execution [12].

**Figure 1:**
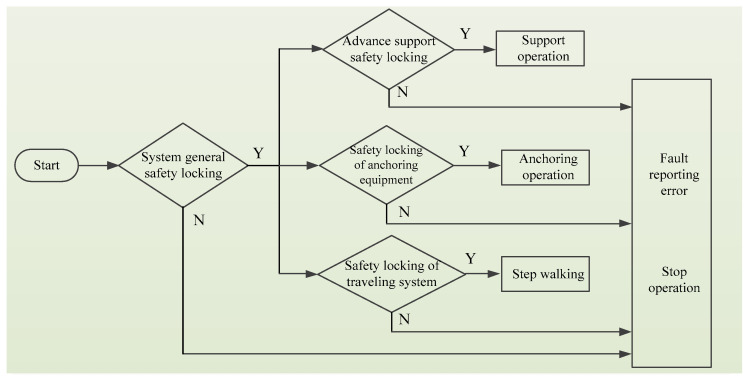
Logical block diagram of the sensing module of the safety interlock system.

Because the combined support and anchor equipment studied herein has the functions of walking, supporting, bolting, etc., the sensor module should comprise monitoring sensors at the advanced support equipment position, the bolter position, and the walking equipment position. The monitoring sensors at the advanced support equipment position are installed at the four corners on the top beam of the support equipment to monitor the distance between the advanced support system and the other sub-equipment or obstacles during movement support. The monitoring sensors at the bolter position are installed on the support base of the jumbolter to monitor the distance between the jumbolter and the surrounding obstacles when the jumbolter is not being operated. The monitoring sensors at the walking equipment position are installed at the four corners of the gantry frame to monitor the distance between the whole machine and the obstacles in the moving and turning process. The logic diagram of the sensor module is shown in Figure 1.

S1: Initialize the safety interlock system of support and anchor equipment.

S2: Judge whether the general safety interlock system of the stepping-type combined support and anchor equipment is locked. If yes, the next step is performed; if no, the system stops running and displays an error report.

S3: Judge whether the safety interlock system of the advanced support equipment is locked. If yes, the next step is performed; if no, the system stops running and displays an error report.

S4: Judge whether the safety interlock system of the bolter is locked. If yes, the next step is performed; if no, the system stops running and displays an error report.

S5: Judge whether the safety interlock system of the walking equipment is locked. If yes, the next step is performed; if no, the system stops running and displays an error report.

## 3. Design of a Logic Control Module for Safety Interlock System

Due to the complex working conditions of the combined support and anchor equipment, the flow demand of the hydraulic system varies greatly, so a single mode of flow control can barely meet the needs of actual operation. This is the reason why this paper adopts pump-valve collaborative control. At present, there are two types of pump-valve collaborative control: the parallel type and tandem type. The parallel type is composed of a relatively independent pump control system and valve control system; the tandem type is composed of control valves in series in the pump control circuit. The parallel type has high system reliability and working efficiency, but two independent hydraulic circuits are needed, so the system structure is complex. The tandem type only requires one oil source, so it has the advantages of simple structure, low cost and high reliability [13]. Considering the mechanical structure and application position of the studied equipment, this paper chooses the tandem-type pump-valve collaborative control system as the control mode of the power system.

### 3.1. Modelling of the Pump Control Part for Pump-Valve Collaborative Control System

This paper chooses to power the system by using a permanent magnet synchronous motor (PMSM) to drive the plunger pump.

#### 3.1.1. Mathematical Model of PMSM in a Natural System of Coordinates

The basic equation of PMSM in a natural system of coordinates is established as follows [14,15]:

(1) $$U=\left[\begin{array}{c} u_{A}\\ u_{B}\\ u_{C} \end{array}\right]=\left[\begin{array}{lll} R & & \\ & R & \\ & & R \end{array}\right]\left[\begin{array}{c} i_{A}\\ i_{B}\\ i_{C} \end{array}\right]+\frac{d}{dt}\left[\begin{array}{c} \psi_{A}\\ \psi_{B}\\ \psi_{C} \end{array}\right]$$

(2) $$\left[\begin{array}{c} \psi_{A}\\ \psi_{B}\\ \psi_{C} \end{array}\right]=\left\{L_{m}\left[\begin{array}{lll} 1 & \cos2\pi /3 & \cos4\pi /3\\ \cos2\pi /3 & \begin{array}{cc} & 1 \end{array} & \cos2\pi /3\\ \cos4\pi /3 & \cos2\pi /3 & \begin{array}{cc} & 1 \end{array} \end{array}\right]+L_{z}\left[\begin{array}{lll} 1 & 0 & 0\\ 0 & 1 & 0\\ 0 & 0 & 1 \end{array}\right]\right\}\left[\begin{array}{c} i_{A}\\ i_{B}\\ i_{C} \end{array}\right]+\psi_{f}\left[\begin{array}{c} \sin\theta_{e}\\ \sin\left(\theta_{e}-2\pi /3\right)\\ \sin\left(\theta_{e}+2\pi /3\right) \end{array}\right]$$

(3) $$T_{e}=\frac{p_{n}}{2}\frac{\partial }{\partial \theta_{m}}\left(\left[\begin{array}{ccc} i_{A} & i_{B} & i_{C} \end{array}\right]\cdot \left[\begin{array}{c} \psi_{A}\\ \psi_{B}\\ \psi_{C} \end{array}\right]\right)$$

where

(4) $$\omega_{e}=p_{n}\omega_{m}$$

(5) $$\theta_{e}=\int \omega_{e}dt=\int p_{n}\omega_{m}dt$$

where *L_m_* is the stator mutual inductance (H); *L_z_* is the stator leakage inductance (H); *ψ_A_*, *ψ_B_*, and *ψ_C_* are the flux linkage of the three-phase winding (Wb); *ψ_f_* is the flux linkage of the fundamental wave of the permanent magnet (Wb); *i_A_*, *i_B_*, and *i_C_* are the current of the three-phase winding, A; *u_A_*, *u_B_*, and *u_C_* are the phase voltage of the three-phase winding (V); *p_n_* is the pair of poles; *R* is the resistance of the three-phase winding (Ω); *θ*_e_ is the rotor electrical angle (rad); *θ_m_* is the mechanical angle (rad); *ω*_e_ is the electrical angular velocity (rad/s); *ω_m_* is the mechanical angular velocity (rad/s); *T_e_* is the electromagnetic torque (N·m).

#### 3.1.2. Mathematical Model of PMSM in a Synchronous Rotating Reference Frame

Under the constraint of constant amplitude, the Clarke transformation and the Park transformation were performed successively to convert the PMSM equation from the natural system of coordinates to the synchronous rotating reference frame *d*-*q*. The coordinate transformation matrix was *T_CP_*:

(6) $${\left[\begin{array}{cc} f_{d} & f_{q} \end{array}\right]}^{T}=T_{CP}{\left[\begin{array}{ccc} f_{A} & f_{B} & f_{C} \end{array}\right]}^{T}=\frac{2}{3}\left[\begin{array}{lll} \cos\theta_{e} & \cos\left(\theta_{e}-\frac{2\pi }{3}\right) & \cos\left(\theta_{e}+\frac{2\pi }{3}\right)\\ -\sin\theta_{e} & -\sin\left(\theta_{e}-\frac{2\pi }{3}\right) & -\sin\left(\theta_{e}+\frac{2\pi }{3}\right)\\ \begin{array}{cc} & \frac{1}{2} \end{array} & \frac{1}{2} & \frac{1}{2} \end{array}\right]{\left[\begin{array}{ccc} f_{A} & f_{B} & f_{C} \end{array}\right]}^{T}$$

(7) $$\{\begin{array}{l} u_{d}=Ri_{d}+\frac{d\psi_{d}}{dt}-\omega_{e}\psi_{q}\\ u_{q}=Ri_{d}+\frac{d\psi_{q}}{dt}+\omega_{e}\psi_{d}\\ \psi_{q}=L_{q}i_{q}\\ \psi_{d}=L_{d}i_{d}+\psi_{f} \end{array}$$

where *u_d_* and *u_q_* are the *d*-*q* shaft voltage (V); *i_d_* and *i_q_* are the *d*-*q* shaft current (A); *ψ_d_* and *ψ_q_* are the *d*-*q* shaft flux linkage (Wb); *L_d_* and *L_q_* are the *d*-*q* shaft inductance (H).

Equation (7) is rewritten into the symmetric matrix equation below:

(8) $$\left[\begin{array}{c} u_{d}\\ u_{q} \end{array}\right]=\left[\begin{array}{ll} R+\frac{d}{dt}L_{d} & -\omega_{e}L_{q}\\ -\omega_{e}L_{q} & R+\frac{d}{dt}L_{d} \end{array}\right]\left[\begin{array}{c} i_{d}\\ i_{q} \end{array}\right]+\left[\begin{array}{c} 0\\ \left(L_{d}-L_{d}\right)\left(\omega_{e}i_{d}-pi_{d}\right)+\omega_{e}\psi_{f} \end{array}\right]$$

The electromagnetic torque (*T*_e_) is:

(9) $$T_{e}=\frac{3p_{n}i_{q}}{2}\left[i_{d}\left(L_{d}-L_{q}\right)+\psi_{f}\right]$$

The output flow *Q_p_* of the hydraulic pump can be expressed as:

(10) $$Q_{p}=nD-K_{c}P_{s}$$

where *n* is the revolving speed of the hydraulic pump (r/min); *D* is the rated displacement of hydraulic pump (L/r); *K*_c_ is the leakage coefficient of the hydraulic pump; *P_s_* is the inlet pressure of the overflow valve (MPa).

The output oil of the pump control system passes the overflow valve to stabilize the system pressure. From the internal flow balance equation of the overflow valve and the balance equation of valve element stress, the transfer function between the inlet pressure of the overflow valve *P_s_* and the overflow quantity *Q_r_* can be obtained [3,11]:

(11) $$\{\begin{array}{l} G(s)=\frac{P_{s}(s)}{Q_{r}(s)}=\frac{m_{R}s^{2}+B_{R}s+K_{R}}{\xi_{p}m_{R}s^{2}+(\xi_{p}m_{R}+AA_{R})s+\xi_{p}K_{R}+\xi_{x}A_{R}}\\ Q_{r}=Q_{p}-Q_{L}\\ A_{R}=A-2c_{d}\omega_{r}x_{0}\cos\theta \\ K_{R}=K_{s}+2c_{d}\omega_{r}P_{s0}\cos\theta \\ B_{R}=A^{2}R\\ \xi_{p}=c_{d}\omega_{r}x_{0}/\sqrt{2\rho P_{s0}}=Q_{s0}/2P_{s0}\\ \xi_{x}=c_{d}\omega \sqrt{2P_{s0}/\rho }=Q_{r0}/x_{0} \end{array}$$

where *m_R_* is the overflow valve element mass (kg); *A_R_* is the equivalent area of overflow valve element (m^2^); *A* is the sectional area of overflow valve element (m^2^); *ω* is the valve port area gradient; *x*_0_ is the spring offset at the balance point (m); *θ* is the intersection angle between the flux axis line and valve element axis line; *K_R_* is the overflow valve spring equivalent stiffness; *Q_r_*_0_ is the steady-state value of overflow quantity; *P_s_*_0_ is the system pressure at the balance point (MPa); *K_s_* is the spring stiffness; *Q_p_* is the hydraulic pump output flow; *Q_L_* is the load flow.

Therefore, the overflow valve inlet pressure can be expressed as:

(12) $$P_{s}(s)=\frac{\left[K_{m}Du_{m}-Q_{L}(\tau_{m}s+1)\right](m_{R}s^{2}+B_{R}s+K_{R})}{K_{L}(\tau_{m}s+1)[\frac{s^{2}}{\omega_{R}^{2}}+\frac{2\xi_{R}s}{\omega_{R}}+1]}$$

where *K_m_* is the motor speed gain coefficient; *τ_m_* is the time constant; *u_m_* is the control voltage signal; *K_R_* is the equivalent stiffness of the overflow valve spring.

### 3.2. Modelling of Valve Control Part of Pump-Valve Collaborative Control System

In order to facilitate analysis of the valve control process of the pump-valve collaborative control system, this paper takes the activity of a hydraulic cylinder as the research object and establishes the relationship equation between input current and output flow [13,16]:

(13) $$\Phi_{sf}(s)=\frac{X_{v}}{I}=\frac{K_{sf}}{\frac{s^{2}}{\omega_{sv}^{2}}+\frac{2\xi_{sv}s}{\omega_{sv}}+1}$$

(14) $$\{\begin{array}{l} Q_{L}=k_{q}x_{v}+k_{c}\left(P_{s}-P_{L}\right)\\ k_{q}=\frac{\partial Q_{L}}{\partial x_{v}}=c_{d}\omega \sqrt{\frac{P_{s}-P_{L}}{\rho }}\\ k_{c}=\frac{\partial Q_{L}}{\partial \left(P_{s}-P_{L}\right)}=\frac{c_{d}\omega x_{v}\sqrt{\frac{1}{\rho }}}{2\sqrt{P_{s}-P_{L}}} \end{array}$$

where *ω_sv_* is the inherent frequency of the valve (rad/s); $\xi_{sv}$ is the damping ratio of the valve; *K_sf_* is the valve’s main spool displacement gain (m/A); *x_v_* is the spool displacement.

Suppose the initial volume of the two cavities of the piston is *V*_10_ = *V*_20_ = *V*_0_, and *A*_1*y*_ ≪ *V*_0_, A_2*y*_ ≪ *V*_0_; then, the flow continuity equation and the force balance equation of the hydraulic cylinder are:

(15) $$\{\begin{array}{l} Q_{L}=A\frac{dy}{dt}+\frac{V_{t}}{4\beta_{e}}\frac{dP_{L}}{dt}+P_{L}C_{tc}\\ A\left(P_{1}-P_{2}\right)=AP_{L}=M\frac{d^{2}y}{dt^{2}}+B_{c}\frac{dy}{dt}+Ky+F \end{array}$$

where *C_tc_* is the total leakage coefficient.

**Figure 2:**
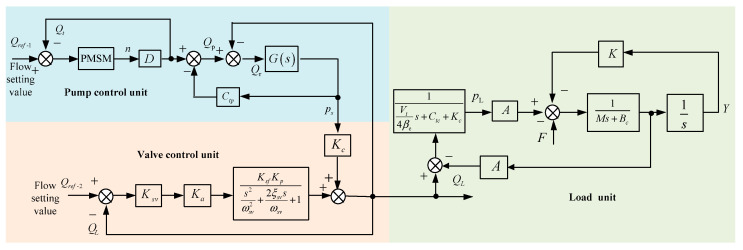
Control block diagram of pump-valve cooperative compound control system.

Figure 2 shows the control block diagram of the established pump-valve cooperative compound control system.

### 3.3. Valve Control System Based on Feedforward–Feedback Optimization

**Figure 3:**
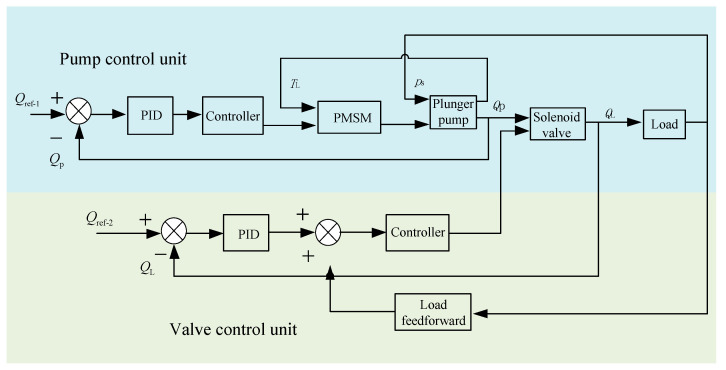
Block diagram of the valve control system based on feedforward–feedback optimization.

General hydraulic control systems have a time lag; that is, when a large deviation between the controlled object and the set value occurs, PID is driven to produce a reasonable adjustment. In a different way, following the compensation principle, a feedforward control system adjusts according to the change in perturbation or the given value. It has control according to the magnitude of the disturbance after the disturbance takes place and before the controlled variable changes to compensate for the impact of the disturbance on the controlled variable. Therefore, in order to improve the stability of the designed safety interlock control system herein, a feedforward control system was introduced to optimize the valve control system, as shown in Figure 3. In Figure 3, the feedforward is inputted to compensate for the steady-state error brought about by the flow change of the object, and the load current feedforward is inputted to compensate for the steady-state error brought about by load disturbance.

## 4. Design of the Executor Module

**Figure 4:**
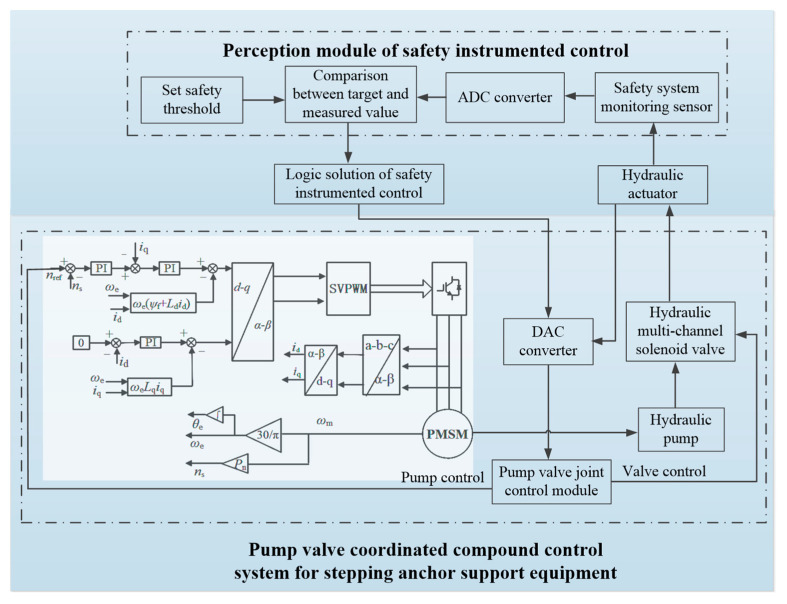
Safety interlock system control principle of combined stepping-type support and anchor equipment.

The logical solution module encompasses signal processing, analysis and judgment, decision output and other functions. First, the information collected by the sensor module is inputted into the logic control circuit through the A/D conversion module for comparative analysis with the preset threshold value. The logic module determines whether to perform walking or turning according to the input information into the logic control circuit, so as to avoid collision between the equipment and other equipment or personnel. At the same time, the safety interlock system of the equipment protects the pump-valve collaborative control system of the power unit to prevent the malfunction or maloperation of the equipment, so as to improve the stability and safety of the equipment. The working principle of the logic solution and executor module is shown in Figure 4.

S1: The signal measured by the sensor module is converted into a digital signal through the A/D conversion module.

S2: The logic control circuit in the logic module is connected to the A/D conversion module to receive the measured data, and then performs amplification, filtering and logical judgment according to the allowable safe value set by the equipment.

S3: The decision signal is transmitted into the electromagnetic valve group and the PMSM driver in the executor module, so as to realize the real-time dynamic control of the pump-valve electrohydraulic system.

S4: The sensor module updates the monitored movement state of the combined stepping-type support and anchor equipment, and repeats the work in steps 1–3.

## 5. Experiment on the Safety Interlock System of Combined Stepping-Type Support and Anchor Equipment

**Figure 5:**
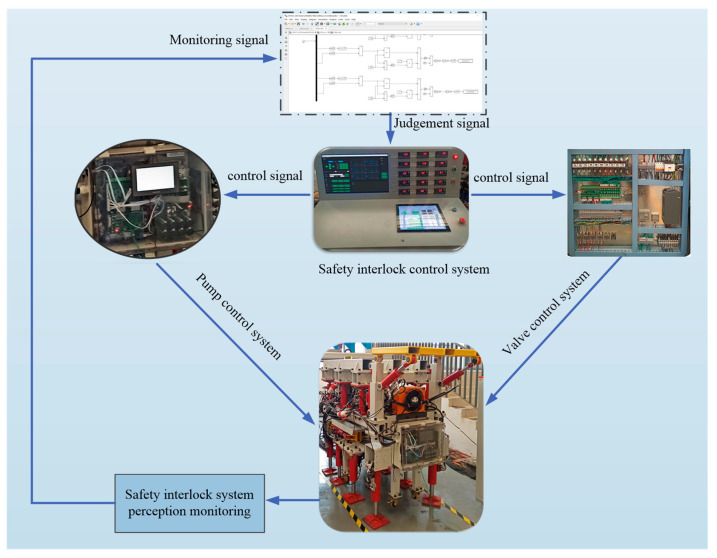
Safety interlock system of combined stepping-type support and anchor equipment.

**Figure 6:**
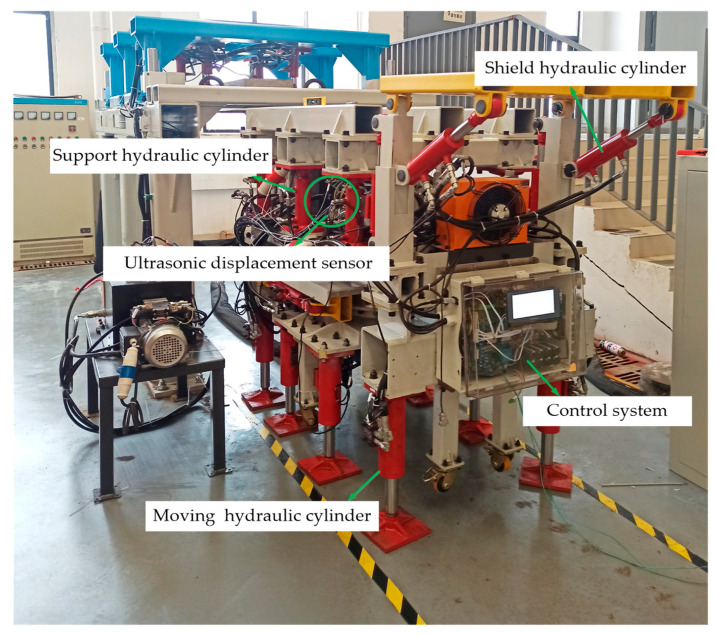
Support and anchor equipment experimental device.

In order to realize the synchronization of the drivage, bolting and supporting processes and improve efficiency, the hardware equipment is constructed according to the control method of the safety interlock system for the combined support and anchor equipment, as shown in Figure 5. The experimental device of support and anchor equipment is shown in Figure 6.

**Figure 7:**
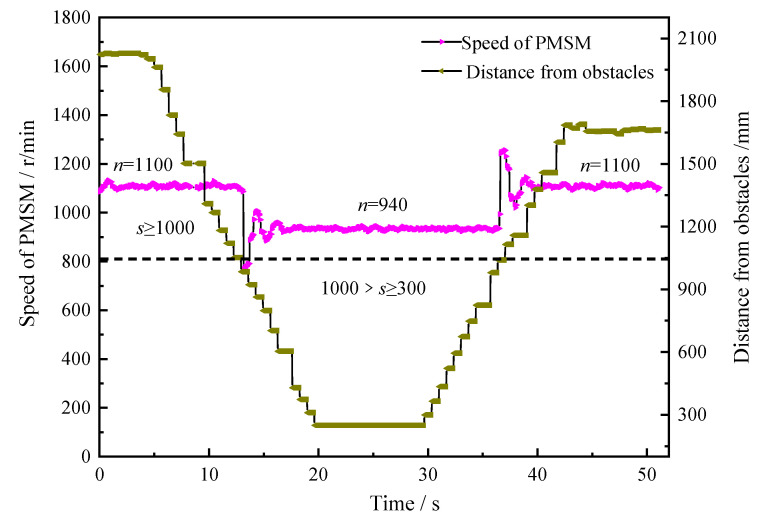
Variation curve of motor speed and system flow controlled by the safety interlocking system.

The safety interlock system of the combined stepping-type support and anchor equipment, shown in Figure 5, first arranges ultrasonic sensor groups at key positions around the equipment to build a virtual working space. When the equipment moves or operates, the safety interlock control system constantly updates the current threshold value, and transmits the control signal to the pump-valve compound control system. An experiment was carried out that targeted the safety interlock control system while the equipment was moving or turning. A three-level control mode was preset according to the distance between the equipment and the obstacle. The experimental results are shown in Figure 7.

According to Figure 7, when the distance from the obstacle (s) is ≥1000 mm, the PMSM operates at a constant speed (ns = 1100 r/min); the moment the distance from the obstacle becomes less than 1000 mm, the revolving speed of the PMSM rapidly drops, fluctuates slightly, and then stabilizes at 940 r/min after 0.2 s; the moment the distance from the obstacle becomes more than 1000 mm, the revolving speed of the PMSM surges to 1100 r/min and remains stable.

**Figure 8:**
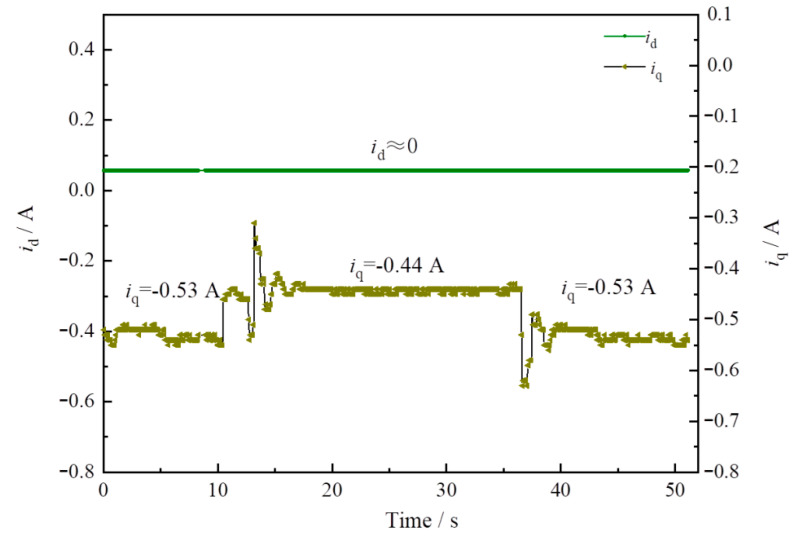
The *i*_d_–*i*_q_ curve of PMSM based on the safety interlock control system.

**Figure 9:**
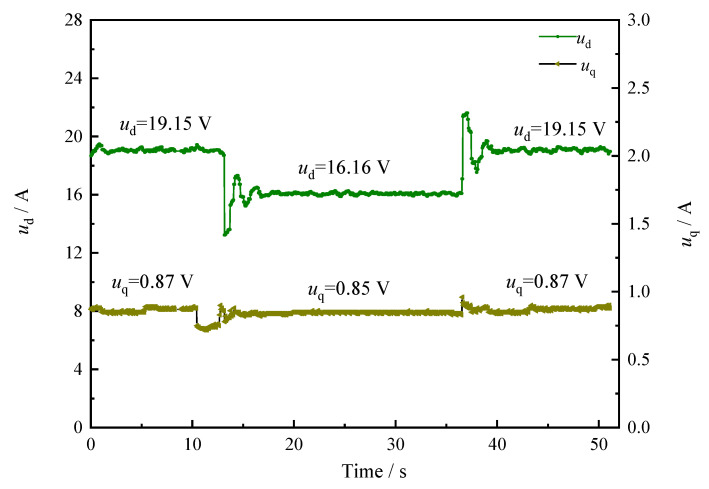
The *u*_d_–*u*_q_ curve of PMSM based on the safety interlock control system.

In the pump control system, the mechanical characteristics of the PMSM are controlled according to Equations (7) and (8). According to Figure 8 and Figure 9, the q-axis current values and the d-axis voltage values of the motor change with the distance between the equipment and the obstacle, and the change law is the same as the motor speed change law—that is, when the distance between the equipment and the obstacle is small, there is a risk of collision, so the control current and voltage of the motor are reduced to protect the equipment from damage.

**Figure 10:**
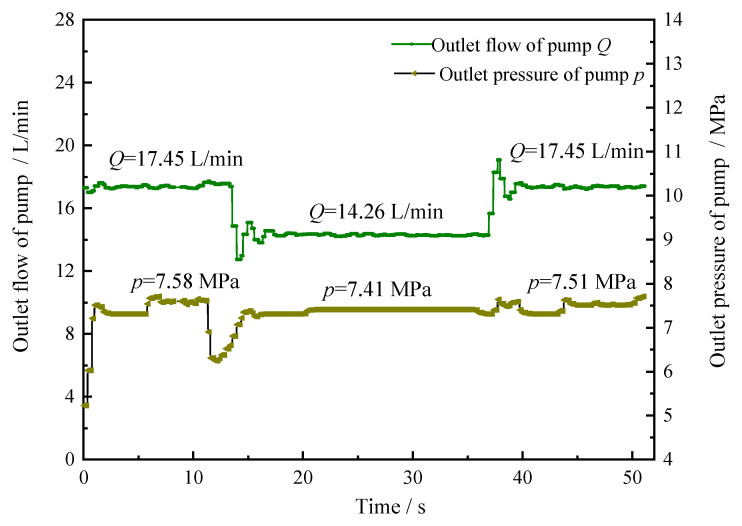
The curve of pressure flow based on safety interlock control system.

**Figure 11:**
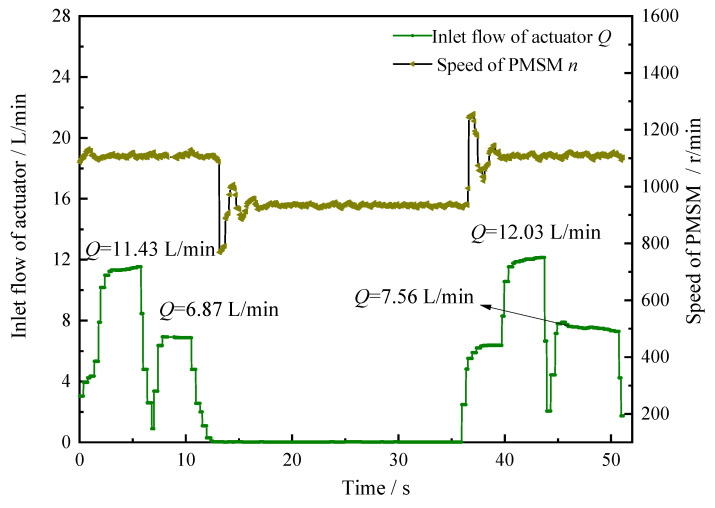
The input flow change curve of actuator based on safety interlock control system.

It can be further seen from the flow change curves of the total pump outlet flow and the actuator in Figure 10 and Figure 11 that the moment the equipment moves to less than 1000 mm from the obstacle, the pump outlet flow decreases from 17.45 L/min to 14.26 L/min within just 0.3 s. At the same time, the system pressure changes accordingly at different stages to prevent equipment malfunction damage to the surrounding obstacles.

## 6. Conclusions

The safety interlock system is an important part of combined support and anchor equipment, which plays a crucial role in ensuring the safety of the equipment in stepping-type movements, supporting and bolting operations. Based on the mobile safe operation requirements for the combined stepping-type support and anchor equipment, this paper presents a safety interlock protection logic diagram, completes the design of a sensor module and a pump-valve collaborative control system for the safety interlock system, establishes an experimental platform for the safety interlock control system of the combined stepping-type support and anchor equipment, and verifies the reliability of the safety interlock system and the closed-loop safety interlock control. This study is of great significance for improving the efficiency of collaborative operation on roadways and reducing the incidence of coal mine accidents.

## Data Availability

Not applicable.
